# Cognitive Exercise Self-Efficacy of Community-Dwelling Older Adults: Measurement and Associations with Other Self-Reported Cognitive Exercise Factors

**DOI:** 10.3390/brainsci11060672

**Published:** 2021-05-21

**Authors:** Therese M. O’Neil-Pirozzi

**Affiliations:** 1 Department of Communication Sciences and Disorders, Northeastern University, 360 Huntington Ave, Boston, MA 02115, USA; t.oneil-pirozzi@northeastern.edu; Tel.: +1-617-373-5750; 2 Department of Physical Medicine and Rehabilitation, Spaulding Rehabilitation Hospital, 300 1st Avenue, Charleston, MA 02129, USA

**Keywords:** challenge, enjoyment, motivation, self-efficacy, cognition, exercise

## Abstract

Exercise self-efficacy, the confidence a person has in their ability to develop and meet exercise goals, is key to exercise motivation. The primary objective of this pilot study was to explore associations among cognitive exercise self-efficacy, cognitive exercise frequency, challenge, and enjoyment in older adults. A prospective, cross-sectional, observational study design was used with 133 community-dwelling individuals aged 55 years and older. Respondents completed a cognitive exercise self-efficacy scale and responded to cognitive exercise queries. Individuals who engaged in cognitive exercise demonstrated greater cognitive exercise self-efficacy. Cognitive exercise self-efficacy ratings were significantly different across challenge and enjoyment conditions (Pearson’s χ^2^ test, *df* = 9, *N* = 133, χ^2^ = 123.49, *p* < 0.01), such that the greater the perception of each, the greater the cognitive exercise self-efficacy (*p* < 0.01). The comparative impact of perceived enjoyment on cognitive exercise self-efficacy was greater than the impact of perceived challenge. Study findings support positive associations among cognitive exercise self-efficacy, cognitive exercise frequency, challenge, and enjoyment. Consideration of these findings may inform design and sustained implementation of motivating cognitive exercise programs to maximize health and quality of life outcomes of healthy and neurologic older adult populations.

## 1. Introduction

Exercise encompasses regular activity that a person engages in to stay healthy or to get healthy, with two types of exercise being physical (e.g., walking every morning) and cognitive (e.g., reading) [1]. Not yet as well studied or understood as physical exercise, cognitive exercise consists of stimulating activities that make a person think and process information and include a variety of activities (e.g., educational or learning, computerized and non-computerized games or puzzles, and musical or creative) [2,3].

While differences exist between physical and cognitive exercise (e.g., primary outcomes by which benefit of each is measured), similarities exist as well. For example, like physical exercise, which requires physical exertion to sustain or improve one’s physical fitness or function, cognitive exercise requires cognitive exertion to sustain or improve one’s cognitive fitness or function [4]. Evidence supports benefits of both physical and cognitive exercise that is regular, repetitive, purposeful, and structured to individuals 55 years of age and older, with and without various medical conditions [1,3,5,6,7]. Despite known benefits of exercise on health, function, and quality of life, initiation of—and persistence with—exercise activity of any kind can be challenging [8,9]. 

Motivation—the need, desire, or willingness of someone to do something to achieve a desired outcome—plays a significant role in the initiation of, and maintained commitment to, any type of exercise activity or program [10]. Multiple theoretical frameworks of motivation and behavioral change exist, with one, social cognitive theory (SCT), having informed this study [11,12,13]. SCT posits that behavioral change is the result of a dynamic and reciprocal interaction among individual, environmental, and behavioral influences. Accordingly, an individual’s motivation to engage in any kind of exercise may be influenced both positively and negatively by internal factors (e.g., previously experienced exercise challenge and enjoyment, metacognition) and by external factors (e.g., education and reinforcement from others). SCT emphasizes that exercise self-efficacy, the confidence a person has in their ability to develop and meet exercise goals, is key to exercise motivation, such that the more an individual believes in their ability to successfully achieve desired exercise outcomes, the more motivated (s)he will be to do so [13,14]. Additionally, the more successful a person is at making a desired change, the more they will believe in their ability to continue making desired change, thus maintaining their motivation. In support of this, decreased physical exercise self-efficacy is associated with decreased motivation to engage in physical exercise in cross-sectional and longitudinal studies with older adults [15,16]. However, no published assessment tool of cognitive exercise self-efficacy exists, and no research has explored whether a similar relationship exists between cognitive exercise self-efficacy and motivation to engage in cognitive exercise. 

Based on SCT evidence that challenge is an internal factor that influences exercise motivation, subsequent exercise, engagement, and functional outcomes, some studies have explored the influence of how an individual’s experience or perception of challenge related to physical exercise may influence their physical exercise self-efficacy. In one study with sedentary young adults who were randomized to high-intensity, moderate-intensity, and no-exercise groups, greater levels of exercise challenge were associated with greater increases in post-exercise self-efficacy; however, for at least some study participants, too much challenge was detrimental [17]. In another study with sedentary older adults, exercise self-efficacy decreased at the end of a structured group program when participants were expected to transition to independent exercising, hypothesized as being perceived as more challenging than exercising in the structured group environment [18]. In the only study exploring the influence of challenge on *cognitive* exercise self-efficacy, middle aged and older adults with persisting moderate-to-severe cognitive impairments following acquired brain injuries endorsed the benefit of cognitive exercises that were challenging on increased cognitive exercise self-efficacy [9]. 

Based on SCT evidence that enjoyment is another internal factor that influences exercise motivation, engagement, and outcomes, some studies have explored the influence of how an individual’s experience or perception of enjoyment related to physical exercise may influence their exercise self-efficacy. In a physical activity study with individuals ages 9 to 17 years, pleasurable experience was noted to be critical to increasing physical activity self-efficacy and subsequent frequency of physical activity [19]. In a physical activity intervention study with sedentary adults (mean age 43 years), greater physical activity enjoyment predicted greater physical activity self-efficacy [20]. Additionally, in a study of predictors of long-term maintenance of physical activity with sedentary older adults, greater enjoyment of physical activity positively mediated physical activity self-efficacy [21]. To our knowledge, except for the study by O’Neil-Pirozzi and colleagues [9] cited above in which participants also endorsed the benefit of cognitive exercises that were enjoyable on cognitive exercise self-efficacy, no studies have explored the influence of enjoyment on *cognitive* exercise self-efficacy.

Metacognition, constituting higher-order mental processes that make awareness of one’s own behavior and thoughts possible [22], is another SCT internal factor that may influence both exercise self-efficacy and motivation to exercise. For example, if a person correctly believes that they are capable of successfully engaging in a cognitive exercise program, research supports that they will: (1) be confident of their ability to do so, (2) be motivated to engage in the exercise program, and (3) achieve exercise program success [23,24]. Although some studies have reported decreased self-awareness of attention and memory abilities by healthy older adults [25,26], a significant body of research suggests that metacognitive abilities of healthy adults do not decline substantially with age [27].

Given the above, the primary purpose of this study was to explore the influence of perceived challenge and enjoyment on cognitive exercise self-efficacy of community-dwelling older adults. We hypothesized that: (1) cognitive exercise self-efficacy would be greater for cognitive exercise that is perceived as challenging versus cognitive exercise that is perceived as non-challenging and (2) cognitive exercise self-efficacy would be greater for cognitive exercise that is perceived as enjoyable versus cognitive exercise that is perceived as non-enjoyable. 

## 2. Materials and Methods

### 2.1. Participants

Community-dwelling individuals aged 55 years and older were invited to participate in this online survey study via recruitment flyers posted at—and distributed online through—area senior community centers and older adult services organizations. While limited demographic data was collected for this pilot study, the participant recruitment sites serve a diverse group of older adults culturally, educationally, and socio-economically. Survey respondents consisted of 133 individuals who possessed the computer literacy skills to complete the survey, confirmed at the start of the survey that they resided in the community, and were 55 years of age or older. Forty-one of the respondents (31%) identified as male and 92 (69%) as female. Respondents ranged in age from 55 years to 93 years (*M* = 68.00, *SD* = 9.3823). This study was pre-approved by the Northeastern University Institutional Review Board for the Protection of Human Subjects in Research (#19-07-03). 

### 2.2. Measures

Because no published cognitive exercise self-efficacy scale exists, the Cognitive Exercise Self-Efficacy Scale (CogEx SES) was created for use in this study (see Appendix A). It was adapted from the Boston Royal Center for Active Lifestyle Interventions Toolbox of Measures Physical Exercise Self-Efficacy Scale, which was modified from Bandura’s Exercise Self-Efficacy Scale [28] and has high internal across-items consistency (Cronbach alpha = 0.88) [15]. Like Neupert and colleagues’ physical exercise self-efficacy scale [28], the CogEx SES consists of nine items that assess how sure a respondent is that (s)he will perform cognitive exercise under different conditions (e.g., when feeling pressure to get things done; when tired). Responses are provided using a 4-point Likert scale (1 = very sure; 2 = pretty sure; 3 = a little sure; 4 = not at all sure). Total scale scores across items range from “9” (representing the greatest cognitive exercise self-efficacy possible) to “36” (representing the poorest cognitive exercise self-efficacy possible). The closer a total scale score is to “9”, the greater the cognitive exercise self-efficacy; the closer a total scale score is to “36”, the poorer the cognitive exercise self-efficacy. As is the Physical Exercise Self-Efficacy Scale, the Flesch Reading Ease score for the CogEx SES is “standard/average”, which corresponds to “Plain English” [29,30]. Unlike Neupert and colleagues’ physical exercise self-efficacy scale directions, which do not include a definition of physical exercise, CogEx SES directions include a definition of cognitive exercise. During the CogEx SES scale development phase, a focus group with eight individuals 55 years of age and older verified comprehension of scale directions, the definition of “cognitive exercise”, and each CogEx SES item. To assess test-retest reliability of the CogEx SES scale, these eight individuals completed the 9-item scale twice, with two weeks between scale completions. Test-retest reliability was high (*r* = 0.92) [31]. While neither the Physical Exercise Self-Efficacy Scale nor the CogEx SES have undergone significant psychometric testing to date, psychometric testing of Bandura’s scale has yielded the following: high internal consistency (Cronbach’s alpha = 0.90), 12-month test-retest reliability (*r* = 0.67), and concurrent criterion-related validity based on self-efficacy being predictive of 1- to 12-month exercise adherence [32,33].

Using the same 4-point Likert scale ratings as above (1 = very sure; 2 = pretty sure; 3 = a little sure; and 4 = not at all sure), two questions regarding cognitive exercise challenge were appended to the CogEx SES: “How sure are you that you will exercise when the cognitive exercise is challenging?” and “How sure are you that you will exercise when the cognitive exercise is not challenging?”. Similarly, two questions regarding cognitive exercise enjoyment were also appended: “How sure are you that you will exercise when the cognitive exercise is enjoyable?” and “How sure are you that you will exercise when the cognitive exercise is not enjoyable?”. The focus group described above also verified comprehension of these four questions. 

### 2.3. Procedure

Individuals interested in study participation went to the study flyer-designated website where they: (1) read the study consent form; (2) clicked on the “accept” button if agreeing to participate; (3) confirmed their eligibility for study participation; and (4) provided requested demographic information. After being given the definition and some examples of cognitive exercise, participants were asked if they engage in cognitive exercise. If an individual responded “yes”, they were then asked to list the five most common cognitive exercises in which they engage. Lastly, participants completed the CogEx SES and challenge and enjoyment questions. Time to respond to all survey prompts ranged from 5 to 15 min, and Qualtrics was the online platform used for anonymous survey completion.

### 2.4. Data Analysis and Statistics

Cronbach’s alpha was used to calculate how closely related the CogExe SES items were as a group. Inter-item correlations of 0.70 and above were considered to represent high internal consistency [34].

Pearson’s Chi-squared test was used to assess the relationship between frequency of self-efficacy ratings (“very sure”, “pretty sure”, “somewhat sure”, and “not at all sure” as categorical variables) and the four conditions related to challenge and enjoyment (“challenging”, “not challenging”, “enjoyable”, and “not enjoyable” as categorical variables). Bonferroni-corrected post-hoc pairwise comparisons were used to: (1) compare the impact of challenging versus non-challenging cognitive exercise on cognitive exercise self-efficacy; (2) compare the impact of enjoyable versus non-enjoyable cognitive exercise on cognitive exercise self-efficacy; (3) determine if the positive impact of perceived enjoyment on cognitive exercise self-efficacy was greater than the positive impact of perceived challenge; and (4) determine if the negative impact of perceived enjoyment on cognitive exercise self-efficacy was greater than the negative impact of perceived challenge. 

All statistical analyses were performed using R Language for Statistical Computing (Version 4.0.0, 2020) [35]. Statistical significance was assumed at *p* = 0.05 level.

## 3. Results

One hundred forty-six individuals initiated online survey completion. One hundred thirty-three (91%) completed all survey queries. Data analysis was completed on the 133 individuals with full survey completion. One hundred five individuals (79%) reported that they engage in cognitive exercise, with the most frequently reported types being reading, crossword puzzles and other word games, and writing.

**Figure 1:**
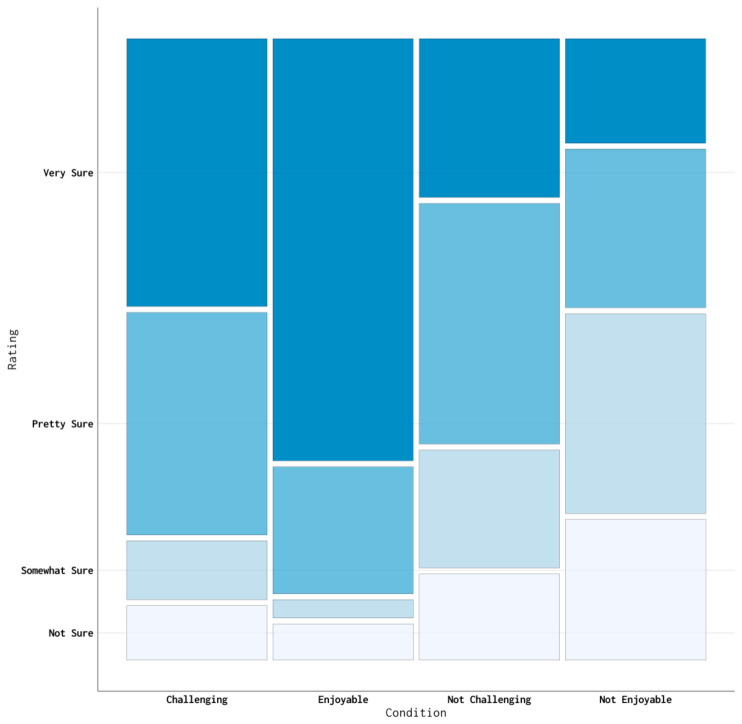
CogEx SES self-efficacy response ratings per condition across study participants.

CogEx SES total scores across the 133 study participants ranged from “9” to ‘‘36”. The median CogEx SES total score was “19”. The most frequent CogEx SES total score was “9”, representing the greatest cognitive exercise self-efficacy possible, by 16 participants (12%). Four participants (3%) had a CogEx SES total score of “36”, representing the poorest cognitive exercise self-efficacy possible. The 105 participants who reported engaging in cognitive exercise had greater cognitive exercise self-efficacy scores than the 28 who did not report cognitive exercise engagement (*t* = 2.03 *df* = 131, *p* = 0.044). Across all challenge and enjoyment conditions, as illustrated in Figure 1, CogEx SES self-efficacy ratings were significantly different (Pearson’s χ^2^ test, *df* = 9, *N* = 133, χ^2^ = 123.49, *p* < 0.01). 

A Cronbach alpha of 0.94 indicated that internal consistency of CogEx SES items was high. This supports that all nine CogEx SES items are measuring the same underlying cognitive exercise self-efficacy construct. 

### 3.1. Impact of Perceived Challenge on Cognitive Exercise Self-Efficacy

Descriptive statistics for each of the challenge questions are summarized in Table 1. Post-hoc Bonferroni-corrected pairwise comparison indicated that cognitive exercise self-efficacy for cognitive exercise that participants perceived as challenging was statistically significantly greater than cognitive exercise self-efficacy for cognitive exercise that they perceived as not challenging (*p* < 0.01). 

### 3.2. Impact of Perceived Enjoyment on Cognitive Exercise Self-Efficacy

Descriptive statistics for each of the enjoyment questions are summarized in Table 2. Post-hoc Bonferroni-pairwise comparison indicated that cognitive exercise self-efficacy for cognitive exercise that participants perceived as enjoyable was statistically significantly greater than cognitive exercise self-efficacy for cognitive exercise that they perceived as not enjoyable (*p* < 0.01). 

### 3.3. Comparative Impact of Perceived Challenge and Enjoyment on Cognitive Exercise Self-Efficacy

The positive impact of cognitive exercise that was perceived as enjoyable on cognitive exercise self-efficacy was greater than the positive impact of cognitive exercise that was perceived as challenging (93/133 versus 59/133 “very sure” ratings, *p* < 0.01). Relatedly, the negative impact of cognitive exercise that was perceived as not enjoyable on cognitive exercise self-efficacy was greater than the negative impact of cognitive exercise that was perceived as not challenging (31/133 versus 19/133 “not at all sure” ratings, *p* < 0.01). 

### 3.4. Gender-Based Challenge and Enjoyment Differences in Cognitive Exercise Self-Efficacy

An unpaired *t*-test of gender-based differences in cognitive exercise self-efficacy for engagement in challenging cognitive exercise revealed no statistically significant gender difference: *t*(131) = 0.2427, *p* = 0.08086. An unpaired *t*-test of gender-based differences in cognitive exercise self-efficacy for engagement in enjoyable cognitive exercise also revealed no statistically significant difference: *t*(131) = 1.9664, *p* = 0.0514. 

## 4. Discussion

Social cognitive theory emphasizes that exercise self-efficacy—the confidence a person has in his or her ability to develop and meet exercise goals—is key to an individual’s motivation to initiate and maintain exercise engagement [13,14]. This association between cognitive exercise self-efficacy and exercise engagement is supported by our study’s finding of greater cognitive exercise self-efficacy scores by those who engaged in cognitive exercise compared with those who did not. Previous studies have positively linked physical exercise self-efficacy and physical exercise outcomes and have examined such influences as challenge and enjoyment on adults’ physical exercise self-efficacy and engagement in physical exercise [17,18,19,20,21]. However, no studies have focused on cognitive exercise self-efficacy and cognitive exercise outcomes, and none have explored their associations with cognitive exercise outcomes. The purposes of this study were to begin filling this knowledge gap by exploring the influence of perceived challenge and enjoyment on cognitive exercise self-efficacy of community-dwelling older adults. 

Our findings support our hypotheses that cognitive exercises perceived as challenging and as enjoyable positively impact older adults’ cognitive exercise self-efficacy. Regarding the comparative impact of challenge and enjoyment, cognitive exercises perceived as enjoyable appear to have a greater positive impact on cognitive self-efficacy than those perceived as challenging. These results are well positioned to inform future related studies and thereby facilitate successful translation of cumulative research findings to benefit older adults’ motivated and sustained engagement in cognitive exercise activity that is associated with positive health and quality of life outcomes. 

### 4.1. Challenge and Cognitive Exercise Self-Efficacy

Consistent with SCT [11,12,28], study participants endorsed the value of challenge on their cognitive exercise self-efficacy and motivation to engage in cognitive exercise. The “challenge” associated with development/selection of challenging cognitive exercise on an individual basis is determining the optimal amount or dose of challenge, with too little or too much challenge potentially compromising exercise self-efficacy and, in turn, impacting exercise engagement and related outcomes [9,17,36]. Adding to this “challenge”, perceptions of what is cognitively challenging vary across individuals based on such factors as education, cognitive ability, and familiarity with/mastery of a specific cognitive activity (e.g., Sudoku) [36,37]. Some research suggests that an individual’s initial degree of exercise challenge be at a mild to moderate level to motivate their initiation of exercise activity and increase their exercise self-efficacy, followed by progressively increased levels of challenge to sustain their exercise engagement and motivation [11,18,38,39]. This study’s findings reinforce the importance of individualized cognitive exercise activities that continuously optimize challenge level based on the ongoing tracking of an individual’s successes and failures so that (s)he ultimately achieves and maintains their targeted cognitive exercise outcomes.

### 4.2. Enjoyment and Cognitive Exercise Self-Efficacy

Additionally, consistent with SCT [11,12,28], study participants strongly endorsed the value of enjoyment on their cognitive exercise self-efficacy and motivation to engage in cognitive exercise. Supported by reports from previous studies regarding enjoyment and exercise-self-efficacy cited earlier in this paper, study findings suggest that individuals are more likely to: (1) believe that they will initiate and maintain engagement in exercise that they enjoy doing more than exercise that they do not enjoy doing and (2) actually initiate and maintain that exercise engagement that they enjoy [9,17,18,19,20,21]. Like challenge, a sense of what is enjoyable is very individualized and may be based on affective feelings (e.g., “doing this exercise is fun.”), on intellectual thoughts and motivations (e.g., “Doing this exercise will help me stay healthy.”, or both (e.g., “Doing this exercise is fun and will help me stay healthy.”). The importance of enjoyment to exercise self-efficacy and exercise engagement further reinforces the importance of individualized cognitive exercise activities to achieve and maintain targeted cognitive exercise outcomes.

### 4.3. Limitations and Future Directions

This is the first study to investigate influences of challenge and enjoyment on cognitive exercise self-efficacy. Thus, additional studies, with a wide range of older adult cohorts and across a variety of settings, are needed. Relatedly, while an online survey is an efficient way to collect data for a study, there is a risk of sample bias related to individuals’ ability to access the Internet.

Except for querying study participants whether or not they engaged in cognitive exercise and, if so, examples of the cognitive exercises that they engaged in, we focused only on relationships between older adults’ perceptions of challenge and enjoyment and their cognitive exercise self-efficacy. Future studies should focus on relationships among older adults’ perceptions of challenge and enjoyment, their cognitive exercise self-efficacy, their motivation, their metacognition, and their documented daily and/or weekly cognitive exercise engagement. For example, does an older adult’s cognitive exercise self-efficacy accurately predict their cognitive exercise engagement? How do an older adult’s metacognitive abilities impact their motivation to engage in challenging versus non-challenging cognitive exercises?

While this initial pilot study did not find statistically significant gender differences in cognitive exercise self-efficacy for engagement in challenging or enjoyable cognitive exercise, this study was not powered to explore these differences explicitly. Especially given the inconsistent findings across studies regarding physical exercise self-efficacy gender differences [40,41] and the absence of previous cognitive exercise self-efficacy gender differences, future studies are needed. Future studies will benefit from collection of additional demographic participant data as well. For example, level of education and socioeconomic status of older adults have been reported to be associated with physical exercise engagement outcomes [42]. Level of education and socioeconomic status of older adults may similarly impact cognitive exercise engagement outcomes, which would reinforce the importance of personalizing cognitive exercise programs to maximize individuals’ motivation, engagement, and outcomes. Cumulatively, this cognitive exercise research trajectory will provide needed evidence to support the health, function, and quality of life of all older adults.

## 5. Conclusions

Positive associations exist between challenge and cognitive exercise self-efficacy and between enjoyment and cognitive self-efficacy in community-dwelling older adults. Translationally, ongoing research of relationships among cognitive exercise self-efficacy, motivational impact of challenge and enjoyment on cognitive exercise goals, and subsequently informed development of personalized cognitive exercise programs may lead to more successful, sustained cognitive exercise engagement by these individuals, which, in turn, may lead to their maximized community-based function.

## Figures and Tables

**Table 1:** Perceived challenge on cognitive exercise self-efficacy scores data summary.

	Number of Participants	Median ^a^	Mode ^a^	Range
Challenging Exercise	133	2	1 (59 times)	3
Non-Challenging Exercise	133	2	2 (53 times)	3

^a^ Response options per question ranged from 1 = very sure will exercise to 4 = not at all sure. The lower the score, the greater the self-efficacy.

**Table 2:** Impact of perceived enjoyment on cognitive exercise self-efficacy scores.

	Number of Participants	Median ^a^	Mode ^a^	Range
Enjoyable Exercise	133	1	1 (93 times)	3
Not Enjoyable Exercise	133	3	3 (44 times)	3

^a^ Response options per question ranged from 1 = very sure will exercise to 4 *=* not at all sure. The lower the score, the greater the self-efficacy.

## Data Availability

The data presented in this study are available upon request from the corresponding author.

## Appendix A. The Cognitive Exercise Self-Efficacy Scale (CogEx SES)

I would like to ask you some questions about cognitive exercise. Cognitive exercise may be defined as a regular activity requiring effort that is carried out to sustain or improve brain health and fitness. Please circle how sure you are that you will do each of the following.

***How sure are you that you will do*** ***each of the following?***	Very Sure	Pretty Sure	A Little Sure	Not At All Sure
Exercise regularly (3 times a week for 20 min)	1	2	3	4
Exercise when you are feeling tired	1	2	3	4
Exercise when you are feeling under pressure to get things done	1	2	3	4
Exercise when you are feeling down or depressed	1	2	3	4
Exercise when you have too much work to do	1	2	3	4
Exercise when there are more interesting things to do	1	2	3	4
Exercise when your family or friends do not provide any kind of support	1	2	3	4
Exercise when you don’t really feel like it	1	2	3	4
Exercise when you are out of your normal routine (e.g., traveling, visiting, on vacation)	1	2	3	4
Exercise when the exercise is challenging	1	2	3	4
Exercise when the exercise is not challenging	1	2	3	4
Exercise when the exercise is enjoyable	1	2	3	4
Exercise when the exercise is not enjoyable	1	2	3	4
Note. Adapted from physical exercise self-efficacy scale in Neupert, Lachman, & Whitbourne 2009 study, available at https://www.brandeis.edu/roybal/docs/Exercise%20Self-Efficacy_website.pdf, accessed on 20 May 2021.				
